# First-in-human radiation dosimetry of the GRPR antagonist [^68^Ga]Ga-NOTA-PEG_2_-RM26 in patients with prostate and breast cancer

**DOI:** 10.1186/s13550-026-01472-2

**Published:** 2026-06-18

**Authors:** Annie Bjäreback, Ulrika Estenberg, Antonios Tzortzakakis, Rimma Axelsson, Chunde Li, Renske Altena, Thuy A. Tran, Cecilia Hindorf

**Affiliations:** 1 Department of Nuclear Medicine and Medical Physics, Section Nuclear Medicine Huddinge, Karolinska University Hospital, Stockholm, Sweden; 2 Department of Molecular Medicine and Surgery, Karolinska Institutet, Stockholm, Sweden; 3 Division of Radiology, Department for Clinical Science, Intervention and Technology (CLINTEC), Karolinska Institutet, Stockholm, Sweden; 4 Department of Oncology-Pathology, Karolinska Institutet, Stockholm, Sweden; 5 Department of Oncology, Södersjukhuset, Stockholm, Sweden; 6 Department of Clinical Science and Education, Karolinska Institutet, Södersjukhuset, Stockholm, Sweden; 7 Department of Nuclear Medicine and Medical Physics, Theranostics Trial Center, Karolinska University Hospital, Stockholm, Sweden

**Keywords:** Radiation dosimetry, First-in-human, Gastrin releasing peptide receptor, GRPR, Positron emission tomography, PET, Prostate cancer, Breast cancer, ^68^Ga

## Abstract

**Background:**

Gastrin-releasing peptide receptor (GRPR) is overexpressed in several malignant tumours, while its physiological expression in major organs, except for pancreas, is low. GRPR is therefore an interesting target in nuclear medicine. The GRPR-binding ligand, [^68^Ga]Ga-NOTA-PEG_2_-RM26 has previously been proven safe in humans. This study evaluates human dosimetry of the radioligand using 12 cancer patients (6 women with breast cancer and 6 men with prostate cancer) based on PET-CT imaging at six timepoints post injection. The voided urine was also collected to serve as an input to a urinary bladder model.

**Results:**

The pancreas, liver, spleen, kidney parenchyma, gall bladder, urinary bladder contents and cortical bone were identified as source organs/regions. Results using OLINDA/EXM 2.3.0 dosimetry software showed that the urinary bladder wall was the organ that received the highest absorbed dose, 197 ± 49 µGy/MBq, followed by the pancreas, 111 ± 42 µGy/MBq, and the kidneys, 32 ± 10 µGy/MBq. The osteogenic cells received 18 ± 5 µGy/MBq. The median (range) of the total excreted activity through urine was 56% (43–75%) of the injected activity in 4h. The effective dose (ICRP 103) from an injection of [^68^Ga]Ga -NOTA-PEG_2_-RM26 was calculated to 19 µSv/MBq, using a 1h voiding interval in the urinary bladder model.

**Conclusion:**

A standard person of 70kg receiving 140MBq of [^68^Ga]Ga-NOTA-PEG_2_-RM26 will receive an effective dose of 2.6 mSv, which is lower or in accordance with other ^68^Ga-labelled GRPR targeted radioligands.

**Trial registration:**

Clinicaltrials.gov, NCT06147362, Registered 17 November 2023 – Retrospectively registered, https://www.clinicaltrials.gov/study/NCT06147362

## Background

Gastrin-releasing peptide receptor (GRPR) is a seven-transmembrane G protein-coupled receptor belonging to the bombesin receptor family [1]. GRPR and its natural ligand gastrin-releasing peptide (GRP) plays an important role in a wide range of physiological functions in the human body [2]. GRPR is predominantly expressed in the pancreas and weakly in the stomach [3].

The GRPR/GRP system has also been observed to have a role in carcinogenesis by its effects on cell growth, differentiation, proliferation and angiogenesis [2, 4]. Accordingly, an overexpression of GRPR has been found in many malignant tumours, for example, prostate, breast, small cell and non-small cell lung cancer and gastrointestinal stromal tumours [1, 5, 6]. The overexpression of GRPR can be utilized as a target for radiolabelled GRPR-binding ligands (radioligands) and is therefore considered an interesting pan-cancer target for radionuclide-based diagnostics and therapy in nuclear medicine [5].

Receptor-specific radioligands can act as agonists or antagonists, determined by their binding characteristics and pharmacological activity [7]. GRPR-binding radioligands were initially developed as agonists, where the internalization of the ligand was considered to prolong tumour uptake. However, agonists were soon replaced by antagonists due to the occurrence of unpleasant, short-term gastrointestinal side effects following administration [8].

Several radiolabelled GRPR antagonist have been investigated for diagnostic imaging in humans; ^68^Ga[Ga]-RM2(BAY 86–7548), ^68^Ga[Ga]-SB3, ^68^Ga[Ga]-NODAGA-MJ9, ^68^Ga[Ga]-NeoBOMB1, ^68^Ga[Ga]-NOTA-PEG_3_-RM26 are some examples [9–13]. The biodistribution of these molecules is similar. The pancreas shows a physiologically high uptake compared to other major organs. The excretion is primarily via the kidneys and to a smaller extent via the small intestine [5] or the hepatobiliary system [9, 14].

All use of ionizing radiation for medical diagnostic purposes, including the administration of radiopharmaceuticals, must be justified, meaning that the risk from the radiation exposure should be weighed against the benefit of the diagnostic procedure [15]. To support justifications, appropriate and robust dosimetry studies are important. Moreover, human dosimetry studies are a requirement from the European Medicines Agency (EMA) and the Food and Drug Administration (FDA) to take new radioligands into further clinical trials [16].

A practical guideline for early phase dosimetry studies was recently published by the European Association of Nuclear Medicine (EANM) [16]. The framework and methodology of the Medical Internal Radiation Dose (MIRD) committee are recommended for internal absorbed dose calculations and the current radiation and tissue weighting factors published by the International Commission on Radiological Protection (ICRP) should be used for radiation safety estimates [15, 17].

Our group has recently published a first-in-human study of the safety and biodistribution of a GRPR-targeting radioligand: [^68^Ga]Ga-NOTA-PEG_2_-RM26 using positron emission tomography (PET) [14]. The result showed a safe compound with the highest physiological uptake in the pancreas, a rapid blood clearance and excretion mainly through the urinary system. However, no human dosimetry data are available for [^68^Ga]Ga-NOTA-PEG₂-RM26. The objective of the present study is to perform dosimetry by calculating the absorbed doses and effective dose of this radioligand.

## Methods

### Patients

Our prospective trial included two patient cohorts: one with hormone-naïve prostate cancer (PC cohort) and one with metastatic estrogen receptor positive (ER +) breast cancer (BC cohort). Complete inclusion and exclusion criteria have previously been published [14]. All participants signed an informed consent of participating in the study that was approved by the Swedish Ethical Review Authority and the Swedish Medical Products Agency (Dnr: 2021–06886-01, EudraCT: 2021–004980-28) and registered on ClinicalTrials.gov (NCT06147362).

### Imaging protocol

**Fig. 1:**
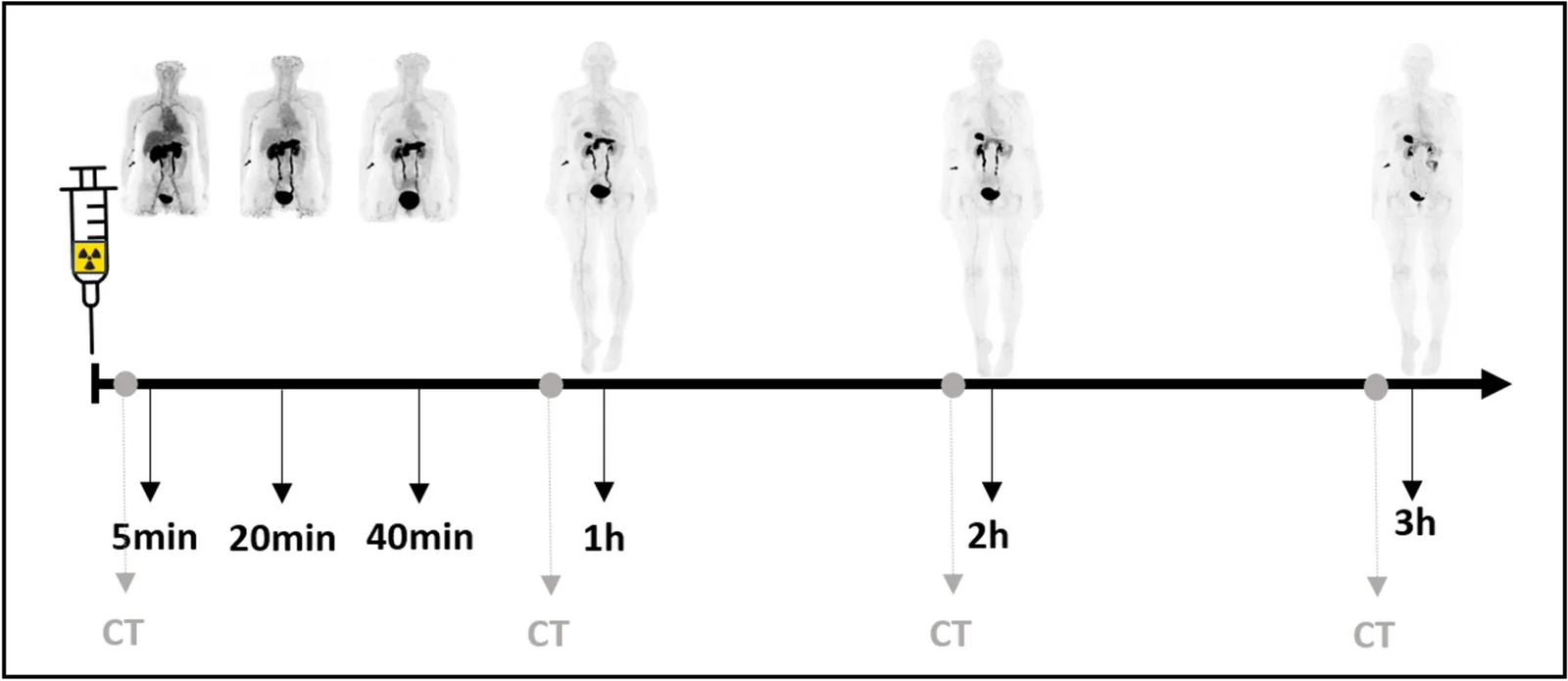
PET-CT scanning protocol. PET scanning was performed from eye level to mid-thigh in the first three scans and from the top of the skull to the toes in the last three scans. In total PET scans from six timepoints after injection were acquired. CT localization were performed just before the 5 min, 1h, 2h and 3h PET scan

The radioligand was produced according to Good Manufacturing Practice (GMP) procedures [18] and imaging was performed on a GE Discovery MI (Milwaukee, WI, USA) positron emission tomography—computed tomography (PET-CT) scanner with four detector rings (20 cm axial field of view). An intravenous injection of 2 MBq/kg of [^68^Ga]Ga-NOTA-PEG_2_-RM26 was given to the participants and PET acquisitions were performed at six time points post injection (5 min, 20 min, 40 min, 1 h, 2 h and 3 h) (Fig. 1). The corresponding acquisition time per bed position was 30 s, 30 s, 1 min, 2 min, 2.5 min, 2.5 min, respectively. The time per bed position was increased to account for radioactive decay but at later timepoints the increase was compromised due to the risk of movement and patient comfort. During the first three time points the scanned field of view ranged from eye level to mid-thigh and scanning was performed sequentially with the patient lying in the same position. In the remaining three time points the scanned field of view was extended to whole body, i.e. from the top of the skull to the feet. Before each of the remaining three time points, the patients were asked to empty their bladder. Whenever the patient was repositioned a localization CT scan was performed (tube voltage 120 kV, noise index 32, pitch 0.984, rotation time 0.5 s), four times in total. The PET images were reconstructed using an ordered-subsets expectation maximization (OSEM) iterative reconstruction algorithm with 3 iterations and 16 subsets followed by 3D gaussian postfiltering (FWHM 5.5 cm). Data were corrected for time-of-flight, attenuation, randoms and scatter.

### Collection of urine

Participants were asked to void at four time points after the injection (right after the 40 min, 1 h, 2 h and 3 h scan). The urine was collected and weighed. Five 1 ml samples were prepared from every urine void. The samples were measured in a gamma counter (Hidex AMG, Hidex Oy, Turku, Finland) using an energy window centred at 511 keV ± 10% and with a 1 min acquisition time per sample. The dead-time factor was kept between 1 and 1.5, whereas in that range the output was linear. The mean ^68^Ga activity concentration of the five samples were used to calculate the total ^68^Ga activity in each void.

The calibration factor for ^68^Ga used in the radionuclide calibrator and the gamma counter were traceable to a secondary standard dose calibrator (Fidelis Secondary Standard RC, Southern Scientific, UK) and the activity was assessed to be within 5% (k = 2, 95% conf. interval).

### Radiation dosimetry

The framework defined by the MIRD committee form the basis for the dosimetry performed [17]. Hermes Affinity viewer 3.0 (Hermes Medical Solutions, Stockholm, Sweden) was used for registration of the PET images and segmentation of the organs. The software used for curve fitting, analytical curve integration and dosimetry calculations was OLINDA/EXM 2.3.0 (Hermes Medical Solutions, Stockholm, Sweden).

### Time activity data

Rigid registration was used to match all PET/CT timepoints. Seven source organs were selected based on the uptake of the radioligand, these were pancreas, liver, spleen, kidney parenchyma, gall bladder, urinary bladder contents and cortical bone.

**Fig. 2:**
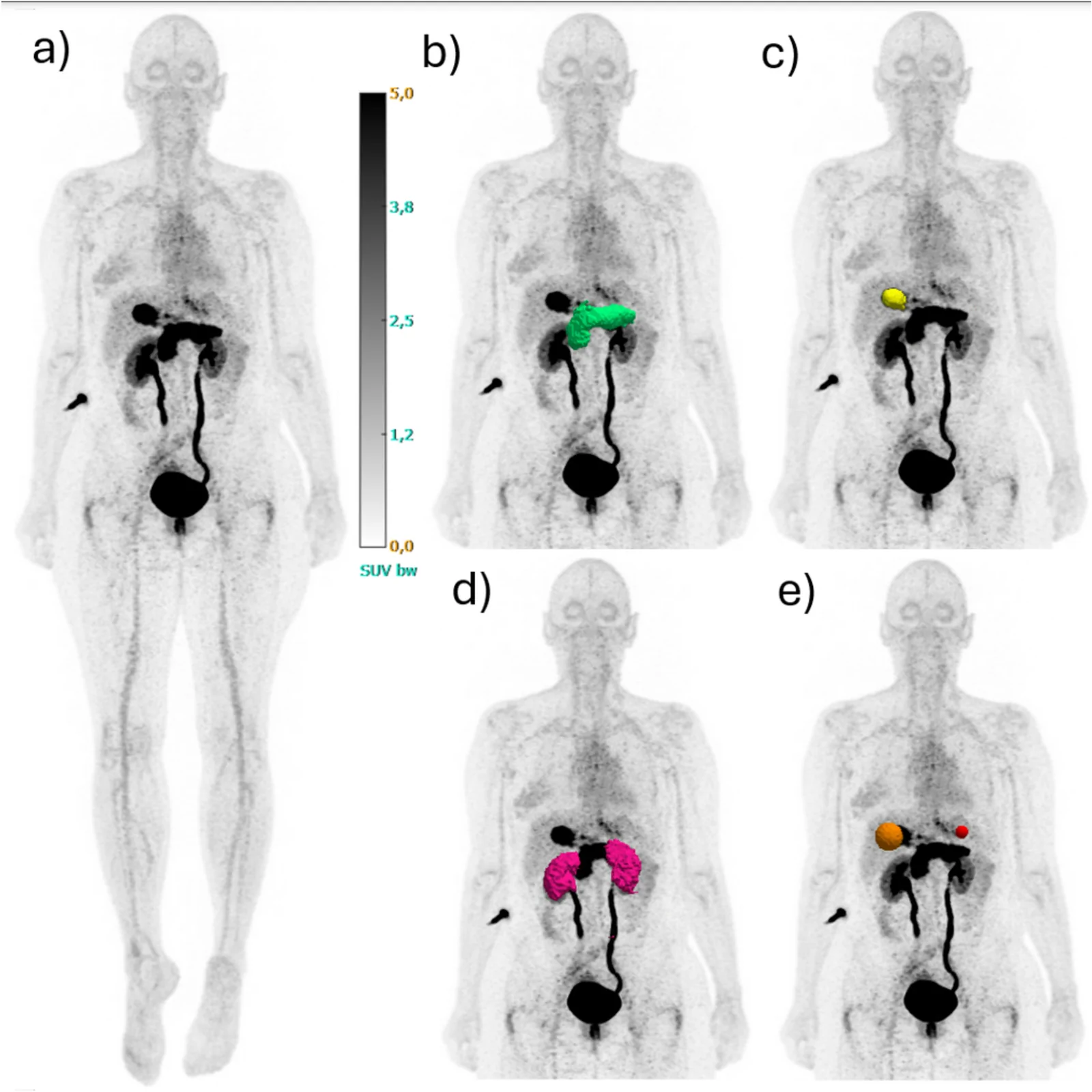
Segmentations of source organs. Maximum intensity projection image of one participant 1h post injection. **a**) Full image, **b**) pancreas segmented, **c**) gall bladder segmented, **d**) kidney cortex segmented, **e**) representative spheres placed in liver and spleen to determine the concentration (Bq/cc)

The methods used for segmentation of source organs differed depending on the appearance of the activity uptake (Fig. 2). The pancreas (Fig. 2b) showed a high and inhomogeneous pattern of PET uptake and therefore the entire organ was segmented by a threshold based (% of Bq/cc) method. The liver and the spleen (Fig. 2e) showed homogenous PET uptake which allowed a concentration-based method; therefore, a representative spherical volume of interest (VOI) was placed in the organ. The activity concentration in the VOI was multiplied by the volume of the organ outlined from the localization CT. The segmentation of the kidney parenchyma was performed by subtracting a VOI of the kidney pelvis from the whole kidney VOI (Fig. 2d). The gall bladder was segmented manually as the activity in the gall bladder differed a lot between timepoints (Fig. 2c). No segmentation was done on the urinary bladder since the time activity data for the urinary bladder content was obtained using the dynamic bladder model [19] implemented in OLINDA/EXM 2.3.0 and data from patient voids were used as input to the model. The voiding interval in the model was set to 1 h.

**Fig. 3:**
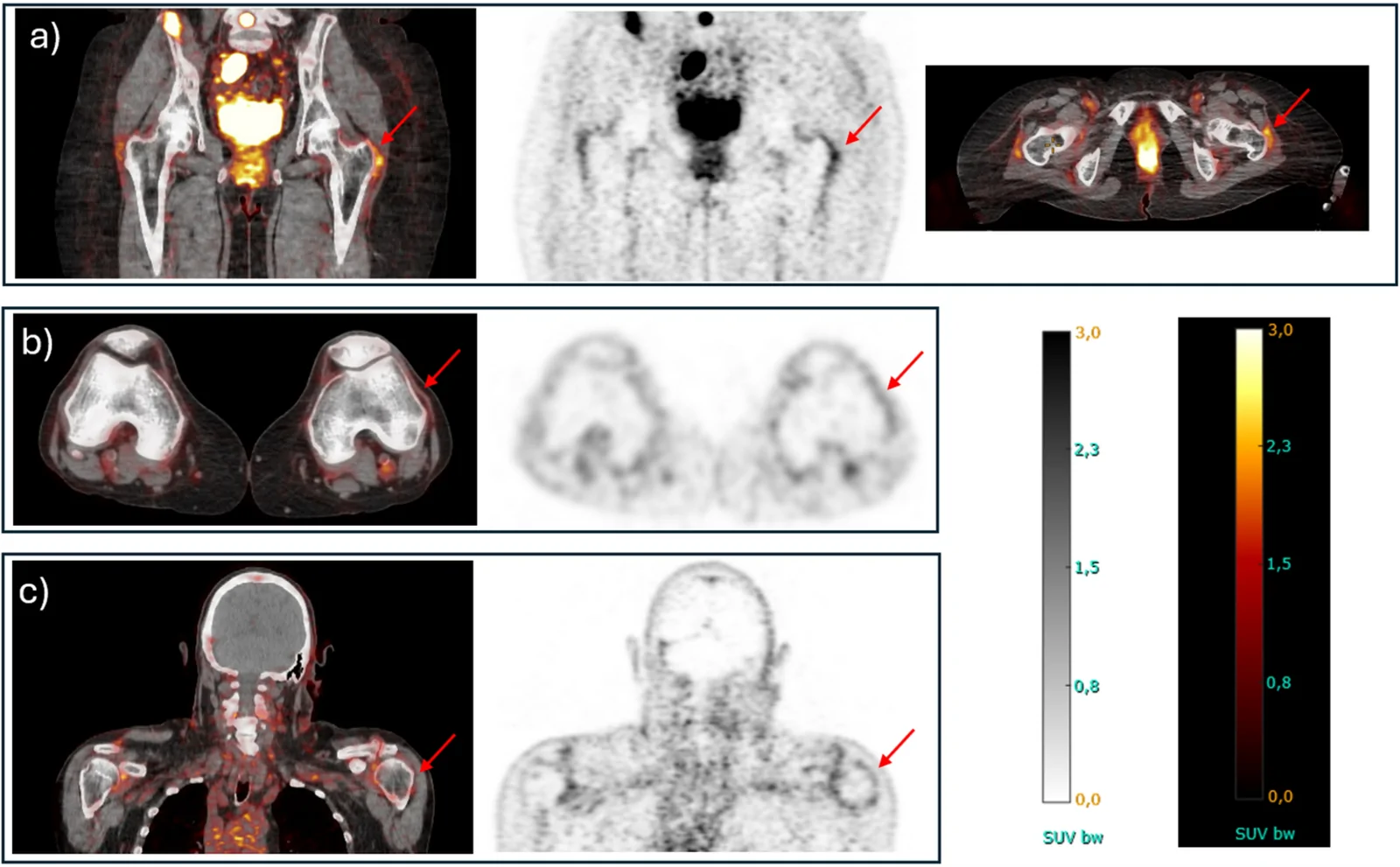
The accumulation/uptake near bone surface in different anatomical locations: **a**) the femur head region in one BC patient, **b**) the lower femur region in one BC patient, **c**) humeral head region in one PC patient

An uptake/accumulation of radioligand could be seen in the vicinity of the bone surfaces e.g. in ligaments and muscle attachments in all participants (Fig. 3). A radiation protection approach was chosen by assigning these uptakes to the surface of the cortical bone in the dosimetry calculation. The femurs including near accumulation of radioligand, not considered a blood vessel, were manually segmented on the PET and CT. The resulting VOI value was extrapolated by scaling the area of the cortical bone in the femur (0.826 m^2^ for both legs) [20] to the area of the cortical bone in the whole skeleton (6.5 m^2^) [20]. Time activity data for this region was only obtained for the last three time points since the complete femur was not included in the first three scans.

The time activity curve for the whole body was based on the injected activity with the urine void activity subtracted. Analytical integration of this curve was performed to obtain the time-integrated activity coefficient (TIAC) for whole body. The remainder was determined by subtracting the sum of the TIAC of the source organs from the whole-body TIAC.

### Time activity curve fitting

Mono- or biexponential analytical curve fitting was performed for the time activity curves of the pancreas, spleen, liver and kidney parenchyma. Goodness of fit was assessed by visual inspection of the fitted curves and by analysis of the coefficient of determination, r^2^. Analytical curve fitting was not used for the gallbladder since it was still in an uptake phase at the 3 h-time point. Trapezoidal integration was therefore used from t = 0 h to t = 3 h. From t = 3 h to infinity, the curve was extrapolated assuming no biological elimination. Trapezoidal integration was also used for cortical bone. In this case the first datapoint was at 1 h post injection so the data was extrapolated with only physical decay to t = 0 h. From 3 h to infinity the curve was extrapolated assuming no biological elimination.

### Absorbed and effective dose calculation

Individual dose calculations for all subjects were performed to capture inter-subject variability and to support descriptive statistical analysis. Since the effective dose is calculated for a reference individual, the mean time-activity data across all patients within each cohort was used to derive time integrated activity coefficients for source organs. The absorbed doses were calculated in using the adult male (for PC cohort) and adult female (for BC cohort) reference phantoms implemented in OLINDA/EXM 2.3.0. with organ weights from ICRP 89 [21]. The equivalent dose, H, was calculated by multiplying the absorbed dose with the most recent radiation weighting factors (w_R_) from ICRP 103 (w_R_ = 1 for γ and β + radiation) [15]. The effective dose, E, was calculated by applying the most recent tissue weighting factors (w_T_) to the sex-averaged equivalent dose for all target organs. The sum of the products results in the ICRP 103 effective dose, E according to the following expression

$$E=\sum_{T}{w}_{T}*\left[\frac{{H}_{T}^{M}+{H}_{T}^{F}}{2}\right]$$

where T is the target tissues, w_T_ is the tissue weighting factor, ${H}_{T}^{M}$ and ${H}_{T}^{F}$ are the male and female equivalent organ doses.

## Results

### Patients

A total of 12 patients, 6 women (median 69 years (range 43–82 years)) diagnosed with metastatic ER + breast cancer and 6 men (median 67 years (range 57–80 years)) diagnosed with prostate cancer were included in the study. The median weight in the BC cohort was 70 kg (range 53–86 kg) and the median weight in the PC cohort was 93 kg (71–113 kg). One of the included BC patients had only one functioning kidney. The same patient had two pancreatic tissues, one native and one transplanted. One BC patient did not have a gall bladder. These two patients were included in the dosimetry calculation despite their anatomical deviations.

### Imaging

A mean activity of 158 ± 27 MBq (1.96 ± 0.15 MBq per kilogram) of [^68^Ga]Ga-NOTA-PEG_2_-RM26 was injected intravenously to the participants. The mean and standard deviation of the PET starting times were 7 ± 1 min, 22 ± 2 min, 40 ± 2 min, 65 ± 4 min, 122 ± 5 min, 178 ± 3 min post injection.

### Urinary samples

Ten participants voided at four time points after injection (54 min ± 5 min, 103 min ± 8 min, 161 min ± 8 min, 218 min ± 7 min). One PC patient voided three times and one BC patient five times. The total amount of activity excreted via the urinary tract for urination number 1, 2, 3, 4 were 24% (8–41%), 14% (10–19%), 10% (6–14%), 9% (9–19%), respectively. Thus, the total excreted activity through urine was 56% (43–75%) in 4 h. Data are reported as median values (range) and decay-correction are applied using the half-life of ^68^Ga (T_1/2_ = 67.83 min [22]).

### Dosimetry

**Fig. 4:**
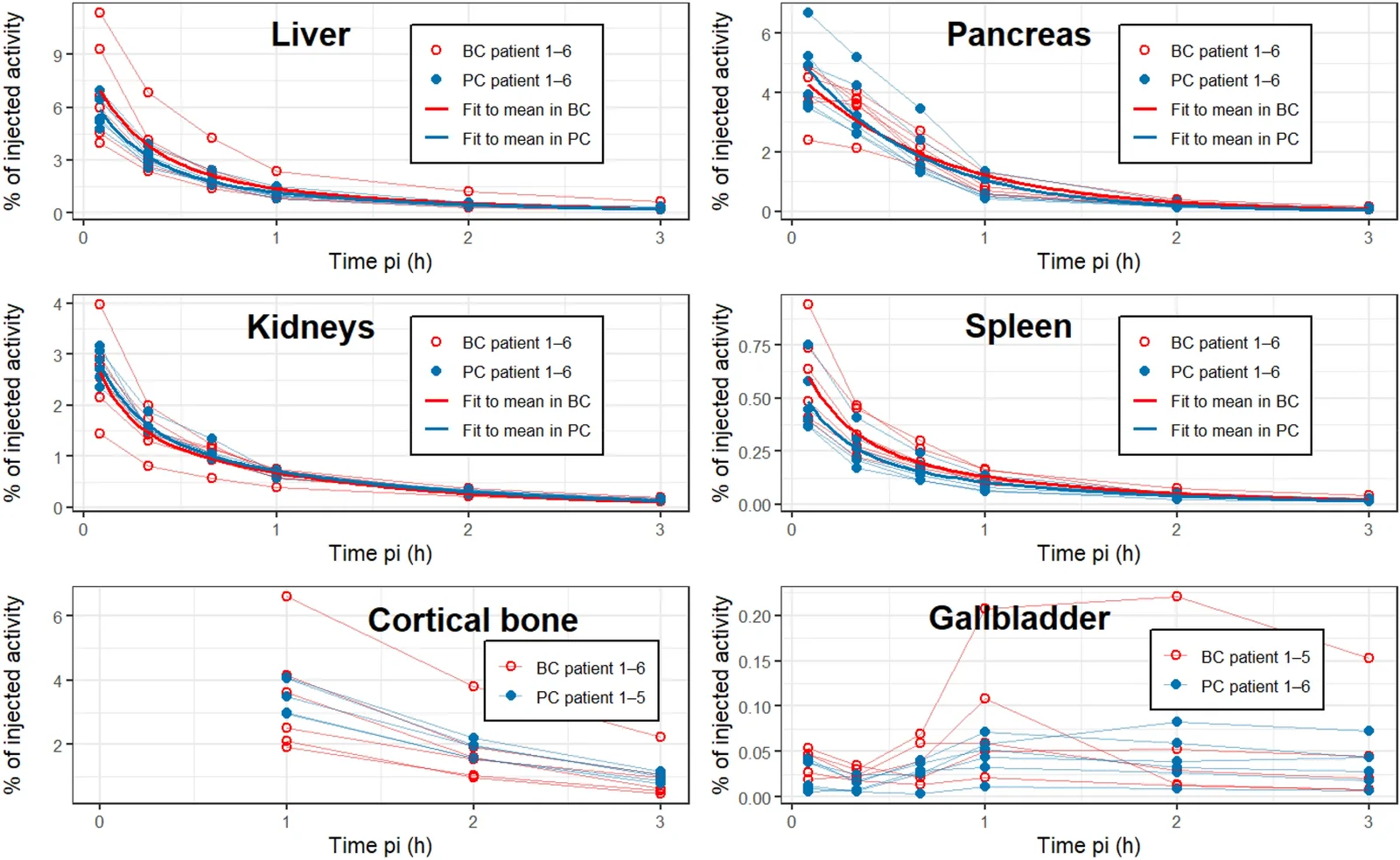
Percent of injected activity as a function of time after injection for six source organs. Data are shown without correction for radioactive decay

Individual time–activity curves for six source organs are shown in Fig. 4. Analytical fits to mean values in each cohort are also shown for all organs, except for cortical bone and gall bladder where trapezoidal integration methods were used.

**Fig. 5:**
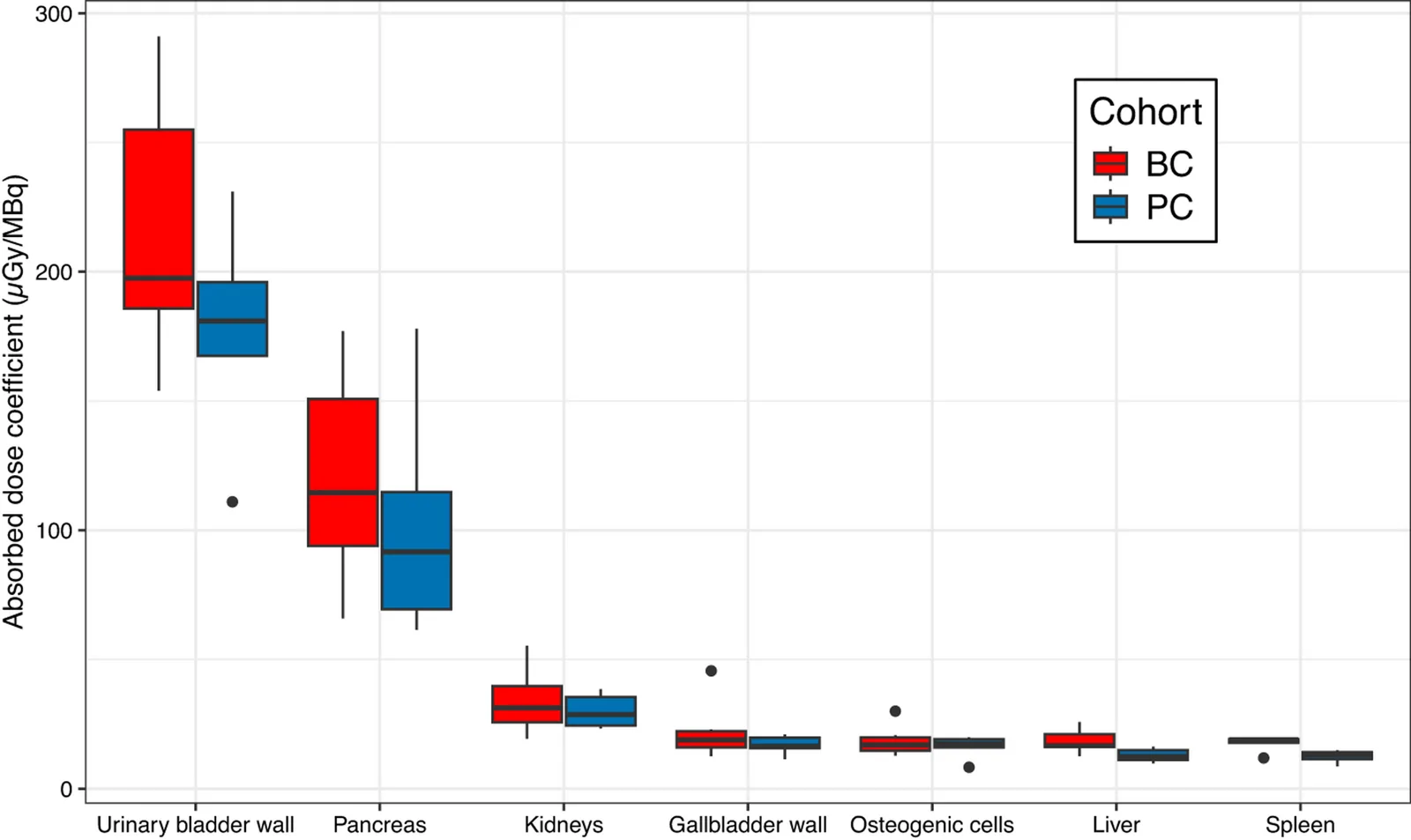
Boxplots showing absorbed dose coefficients (µGy/MBq) for the organs with the highest absorbed dose per injected activity

The urinary bladder wall was the organ that received the highest absorbed dose coefficient, 197 ± 49 µGy/MBq ( BC cohort 216 ± 54 µGy/MBq and PC cohort 178 ± 40 µGy/MBq) (Fig. 5). The pancreas and the kidneys were the organs that received the second- and third-highest doses. The pancreas received 111 ± 42 µGy/MBq (BC cohort 120 ± 42 µGy/MBq, PC cohort 101 ± 44 µGy/MBq). The kidneys received 32 ± 10 µGy/MBq (BC cohort 34 ± 13 µGy/MBq, PC cohort 30 ± 7 µGy/MBq). The absorbed dose per injected activity to the osteogenic cells was 18 ± 4 µGy/MBq (BC cohort 19 ± 6 µGy/MBq, PC cohort 16 ± 4 µGy/MBq) (Table 1). No clear differences in absorbed dose were observed between the cohorts. The values above are reported as mean ± 1 standard deviation from the individual dosimetry calculations.

**Table 1:** Comparison of dosimetry studies of GRPR targeting radioligands

	[^68^ Ga]Ga-NOTA-PEG_2_-RM26 This study	[^68^ Ga]Ga-NOTA-PEG_3_-RM26 (13)	[^68^ Ga]Ga-RM2 (BAY 86–7548) (9)	[^68^ Ga]Ga-NODAGA-MJ9 (11)	[^68^ Ga]Ga-SB3 (10)	[^68^ Ga]Ga-NeoBOMB1 (12)
Nr of individuals (♂/♀)	**12(6/6)**	5(3/2)	5(5/0)	5(5/0)	10(10/0)	6(2/4)
Imaging time points after injection (min p.i.)	**5,20,40,60,120,240**	5,15,30,45, 60	1,10,20,40,100,200	10,45,70,100	7 scans up to 210	5,12,19,60,120,240
Dosimetry software	**OLINDA/EXM 2.3**	OLINDA/EXM 1.1	OLINDA/EXM 1.1	OLINDA/EXM 2.0	IDAC dose 2.1	OLINDA/EXM 1.1
Tissue weighting factors used (w_T_)	**ICRP-103**	ICRP-60	ICRP-60	ICRP-103	ICRP-103	ICRP-60
Bladder model used Y/N (voiding interval)	**Yes (1 h)**	Yes (1 h)	Yes (3.5 h)	Yes (1 h)	Yes (1 h)	Yes (2 h)
**Absorbed dose per injected activity (µGy/MBq)**						
Pancreas	**111**	225	510	260	198	274
Urinary bladder wall	**197**	1090	610	70	116	165
Kidneys	**32**	36	81	35	53	52
Osteogenic cells	**18**	10	13	8	Not specified	18
**Effective dose coefficent (µSv/MBq)**						
	**19**	66	38	22	14	29

The effective dose per injected activity was determined to 19 µSv/MBq for a reference individual. Individual dose calculations showed a median value of 18 µSv/MBq with a range of 14–22 µSv/MBq. The upscaled effective dose for an injected activity of 140 MBq in a 70 kg patient is 2.6 mSv.

## Discussion

In this study, human dosimetry of the new radioligand [⁶⁸Ga]Ga-NOTA-PEG₂-RM26 was evaluated. Twelve patients underwent serial PET/CT examinations after intravenous injection. The urine was collected and measured in a gamma counter. Results showed that the three most irradiated organs were the urinary bladder wall, the pancreas and the kidneys. The effective dose was calculated to 2.6 mSv for a standard person of 70 kg that receives 2 MBq/kg (19 µSv/MBq).

The effective dose, defined by the ICRP, is a radiation protection quantity that approximates the stochastic health risk from a radiation exposure. It is routinely used in clinical practice in justification of the medical use of ionizing radiation. Consequently, dosimetry studies for novel diagnostic radioligands, where effective dose is calculated, play a critical role in assessing and optimizing radiation protection.

Due to a high renal excretion of the radioligand, high activity amounts were accumulated in the urinary bladder. An effect from this was a halo artefact around the urinary bladder that limited the detectability in the pelvic region. These artefacts had however a small impact on the dosimetry calculation since urinary samples were used instead of image-based data to quantify bladder contents. In one patient, the femur head was hidden by the halo and therefore the segmentation of the femur bone was not possible. A decision was made to exclude cortical bone as a source organ in that patient.

A limitation of the present study is the absence of recovery correction to account for partial volume effects (PVE). PVE is particularly pronounced in small volumes of interest (VOIs), where activity underestimation can occur due to limited spatial resolution in the PET system. However, as the segmentation was done manually compensation was done to some extent for the spill out activity. Apart from manual compensation most source organs in this study had a size that was limited influenced by PVE.

Zhang et al. (13) investigated a similar molecule, [^68^Ga]Ga-NOTA-PEG_3_-RM26 with a different PEG spacer length and reported an effective dose per injected activity of 66 ± 12 µSv/MBq which is 3.5 times higher than the value presented in this study (Table 1). The absorbed dose to the urinary bladder wall was also notably higher, 1090 ± 225 µSv/MBq compared to 197 ± 49 µSv/MBq in this study, both calculated with a 1h bladder voiding interval. The absorbed dose to pancreas was 225 ± 38 µGy/MBq compared to 111 ± 42 µGy/MBq in this study.

There are several methodological differences between the two studies which may account for the discrepancies in the reported results. Firstly, the radioligand was observed during a longer time span in the present study (3 h) compared to Zhang et al. [13] (1 h). The shorter observation period requires extrapolation over a larger portion of the time-activity curve (TAC), which may have introduced greater uncertainty and contributed to the differences in dose estimates. Secondly, Zhang et al. used OLINDA/EXM version 1.0 versus version 2.3 in this study. The different version uses different reference phantoms and a different set of tissue weighting factors (ICRP103 in version 2.3 compared to ICRP60 in version 1.0). By applying the tissue weighting factors from ICRP-60 to our data a 21% increase of effective dose could be seen. Thirdly, the study populations differed. Zhang et al. based their dosimetry on 5 healthy volunteers, while the present study included 12 patients with cancer. However, in this study, the biodistribution of the radioligand in normal organs is unlikely to be affected by the presence of malignancies. Therefore, difference in study populations is not considered a major contributor to the observed variation in effective dose.

Other dosimetry studies of ^68^Ga labelled GRPR antagonists are in closer agreement regarding the effective dose (Table 1). For example, Gnesin et al. that investigates [^68^Ga]Ga-NODAGA-MJ9 [11], reports an effective dose coefficient of 22 µSv/MBq and for [^68^Ga]Ga-SB3, Bakker et al. [10] reports 14.4 µSv/MBq both calculated with a 1h voiding interval. Possible explanations for the closer agreement include the use of ICRP 103 weighting factors and that the observation period is more similar to ours, imaging up to 100 min in the study of Gnesin et al. [11] and up to 210 min in the study of Bakker et al. [10].

The voiding interval chosen for the dynamic bladder model was 1 h which could be considered as a relatively short interval. However, this interval was chosen for the dosimetry calculation since it is the most commonly reported interval among the studies in. The effect of absorbed and effective dose from different voiding intervals have been published by Gnesin et al. [11]. According to their findings, an increase of the voiding interval from 0.5 h to 3.5 h increases the absorbed dose to the bladder wall by 186%. The effective dose was increased by 33%.

The primary focus of a dosimetry study for a novel diagnostic radioligand is radiation safety. Given that the bone surface contains radiation-sensitive cells, it was considered important to account for the accumulation of activity observed in close proximity to the bone surface in this study. Segmentation of the entire skeleton was not considered feasible due to spill-over effects from nearby organs with high uptake. Instead, the femurs of both legs were segmented, and the observed uptake was assigned to the cortical bone and extrapolated to the whole skeleton. This extrapolation approach was adopted from the work of Chittenden et al. [23]. We acknowledge that this methodology is conservative and may result in an overestimation of bone surface dose; however, this was deemed appropriate in the context of radiation protection and safety assessment. The osteogenic cells received an absorbed dose per injected activity of 18 µGy/MBq. This value is in accordance with other studies (Table 1) and is low. The inclusion of the cortical bone as a source organ had little effect on the effective dose value (data not shown).

The obtained effective dose for a 70 kg patient from an injection of the studied radioligand was 2.6 mSv and is low compared to other ^68^Ga-labelled receptor-targeting tracers. The corresponding value for [^68^Ga]Ga-PSMA-11 was 3 mSv [24] and for [^68^Ga]Ga-DOTATATE 3.6 mSv [25] (calculated with 2 MBq/kg). As a comparison an injection of 3 MBq/kg of [^18^F]FDG is associated with an effective dose of 4 mSv [26].

## Conclusion

This study aimed to do a thorough dosimetry study of a new GRPR antagonist [^68^Ga]Ga-NOTA-PEG_2_-RM26. The effective dose per injected activity were 19 µSv/MBq resulting in 2.6 mSv for a 70 kg person. This value is lower or in accordance with other GRPR targeted radioligands.

## Data Availability

The datasets generated and analysed during the current study are available from the corresponding author on reasonable request.

## Abbreviations

BC: Breast cancer
CT: Computed tomography
EANM: European association of nuclear medicine
EMA: European medicines agency
ER +: Estrogen receptor positive
FDA: Food and drug administration
^68^Ga: Gallium 68
GRPR: Gastrin releasing peptide receptor
ICRP: International commission on radiological protection
MIRD: Medical internal radiation dose
PC: Prostate cancer
PET: Positron emission tomography
PI: Post injection
PVE: Partial volume effect
TAC: Time activity curve
TIAC: Time integrated activity coefficient
VOI: Volume of interest

## References

[1] Baratto L, Duan H, Mäcke H, Iagaru A. Imaging the distribution of gastrin-releasing peptide receptors in cancer. J Nucl Med. 2020;61(6):792–8. 10.2967/jnumed.119.234971 32060215

[2] Majumdar ID, Weber HC. Biology of mammalian bombesin-like peptides and their receptors. Curr Opin Endocrinol Diabetes Obes. 2011;18(1):68–74. 10.1097/MED.0b013e328340ff93 21042212

[3] Sano H, Feighner SD, Hreniuk DL, Iwaasa H, Sailer AW, Pan J, et al. Characterization of the bombesin-like peptide receptor family in primates. Genomics. 2004;84(1):139–46. 10.1016/j.ygeno.2004.01.008 15203211

[4] Xiao L, Fang Z, Tang Y, Sun Y, Zhu Z, Li J, et al. Evaluation of gastrin-releasing peptide receptor, prostate-specific membrane antigen, and neurotensin receptor 1 as potential biomarkers for accurate prostate cancer stratified diagnosis. EJNMMI Res. 2024;14(1):55. 10.1186/s13550-024-01116-3 PMC11180645 38880858

[5] Morgat C, Duan H, Dalm S, Hindié E, Günther T, Krause BJ, et al. A Vision for gastrin-releasing peptide receptor targeting for imaging and therapy: perspective from academia and industry. J Nucl Med. 2025;66(8):1160–7. 10.2967/jnumed.124.269444 40341094

[6] Mattei J, Achcar RD, Cano CH, Macedo BR, Meurer L, Batlle BS, et al. Gastrin-releasing peptide receptor expression in lung cancer. Arch Pathol Lab Med. 2014;138(1):98–104. 10.5858/arpa.2012-0679-OA 24377816

[7] Nock BA, Kanellopoulos P, Joosten L, Mansi R, Maina T. Peptide radioligands in cancer theranostics: agonists and antagonists. Pharmaceuticals (Basel). 2023;16(5):674. 10.3390/ph16050674 PMC10222684 37242457

[8] Mansi R, Nock BA, Dalm SU, Busstra MB, van Weerden WM, Maina T. Radiolabeled bombesin analogs. Cancers (Basel). 2021;13(22):5766. 10.3390/cancers13225766 PMC8616220 34830920

[9] Roivainen A, Kähkönen E, Luoto P, Borkowski S, Hofmann B, Jambor I, et al. Plasma pharmacokinetics, whole-body distribution, metabolism, and radiation dosimetry of 68Ga bombesin antagonist BAY 86–7548 in healthy men. J Nucl Med. 2013;54(6):867–72. 10.2967/jnumed.112.114082 23564761

[10] Bakker IL, Fröberg AC, Busstra MB, Verzijlbergen JF, Konijnenberg M, van Leenders G, et al. GRPr Antagonist (68)Ga-SB3 PET/CT imaging of primary prostate Cancer in therapy-naïve patients. J Nucl Med. 2021;62(11):1517–23. 10.2967/jnumed.120.258814 PMC8612327 33789933

[11] Gnesin S, Cicone F, Mitsakis P, Van der Gucht A, Baechler S, Miralbell R, et al. First in-human radiation dosimetry of the gastrin-releasing peptide (GRP) receptor antagonist (68)Ga-NODAGA-MJ9. EJNMMI Res. 2018;8(1):108. 10.1186/s13550-018-0462-9 PMC6291411 30543050

[12] Gruber L, Jiménez-Franco LD, Decristoforo C, Uprimny C, Glatting G, Hohenberger P, et al. MITIGATE-NeoBOMB1, a Phase I/IIa study to evaluate safety, pharmacokinetics, and preliminary imaging of (68)Ga-NeoBOMB1, a gastrin-releasing peptide receptor antagonist. GIST Patients J Nucl Med. 2020;61(12):1749–55. 10.2967/jnumed.119.238808 32332143

[13] Zhang J, Niu G, Fan X, Lang L, Hou G, Chen L, et al. PET using a grpr antagonist (68)Ga-RM26 in healthy volunteers and prostate Cancer patients. J Nucl Med. 2018;59(6):922–8. 10.2967/jnumed.117.198929 PMC6004560 29123014

[14] Bjäreback A, Jonmarker O, Tzortzakakis A, Jussing E, Li C, Altena R, et al. First-in-human experience with GRPR antagonist [(68)Ga]Ga-NOTA-PEG(2)-RM26 in prostate and breast cancer patients using PET/CT. EJNMMI Res. 2025;15(1):12. 10.1186/s13550-025-01204-y PMC11842642 39976803

[15] ICRP. The 2007 Recommendations of the international commission on radiological protection. ICRP Publication. 2007;103. 10.1016/j.icrp.2007.10.003 18082557

[16] Stokke C, Gnesin S, Tran-Gia J, Cicone F, Holm S, Cremonesi M, et al. EANM guidance document: dosimetry for first-in-human studies and early phase clinical trials. Eur J Nucl Med Mol Imaging. 2024;51(5):1268–86. 10.1007/s00259-024-06640-x PMC10957710 38366197

[17] Bolch WE, Eckerman KF, Sgouros G, Thomas SR. MIRD pamphlet No. 21: a generalized schema for radiopharmaceutical dosimetry–standardization of nomenclature. J Nucl Med. 2009;50(3):477–84. 10.2967/jnumed.108.056036 19258258

[18] Jussing E, Moein MM, Tegnebratt T, Semetaite V, Liepins E, Tzortzakakis A, et al. P-271 - Preparation and cGMP-compliant production of the bombesin analogue [68Ga]Ga-NOTA-RM26 for first-in-human clinical study of gastrin-releasing peptide receptor expression in prostate Cancer. Nucl Med Biol. 2022;108–109:S197–8.

[19] Thomas SR, Stabin MG, Chen CT, Samaratunga RC. MIRD pamphlet No. 14 revised: a dynamic urinary bladder model for radiation dose calculations. task group of the mird committee, society of nuclear medicine. J Nucl Med. 1999;40(4):102s-s123. 10210232

[20] ICRP. Basic anatomical & physiological data for use in radiological protection - The Skeleton. ICRP Publication 70. Annals of the ICRP. 1995;25. 8659813

[21] Basic anatomical and. physiological data for use in radiological protection: reference values. A report of age- and gender-related differences in the anatomical and physiological characteristics of reference individuals. ICRPPublication 89. Ann ICRP. 2002;32(3–4):5–265. PMID:14506981. 14506981

[22] Becquerel LNH. Table de Radionucleides. 1998.

[23] Chittenden SJ, Hindorf C, Parker CC, Lewington VJ, Pratt BE, Johnson B, et al. A Phase 1, Open-label study of the biodistribution, pharmacokinetics, and dosimetry of 223Ra-dichloride in patients with hormone-refractory prostate cancer and skeletal metastases. J Nucl Med. 2015;56(9):1304–9. 10.2967/jnumed.115.157123 26182965

[24] Sandgren K, Johansson L, Axelsson J, Jonsson J, Ögren M, Ögren M, et al. Radiation dosimetry of [(68)Ga]PSMA-11 in low-risk prostate cancer patients. EJNMMI Phys. 2019;6(1):2. 10.1186/s40658-018-0239-2 PMC6328430 30631980

[25] Walker RC, Smith GT, Liu E, Moore B, Clanton J, Stabin M. Measured human dosimetry of 68Ga-dotatate. J Nucl Med. 2013;54(6):855–60. 10.2967/jnumed.112.114165 PMC4472480 23516312

[26] ICRP. Radiation dose to patients from radiopharmaceuticals - Addendum 3 to ICRP publication 53, ICRP publication 106. Ann ICRP. 2008;38:(1–2). 10.1016/j.icrp.2008.08.003 19154964

