# Correction: First-in-human radiation dosimetry of the GRPR antagonist [^68^Ga]Ga-NOTA-PEG2-RM26 in patients with prostate and breast cancer

**DOI:** 10.1186/s13550-026-01541-6

**Published:** 2026-11-09

**Authors:** Annie Bjäreback, Ulrika Estenberg, Antonios Tzortzakakis, Rimma Axelsson, Chunde Li, Renske Altena, Thuy A. Tran, Cecilia Hindorf

**Affiliations:** 1 Department of Nuclear Medicine and Medical Physics, Section Nuclear Medicine Huddinge, Karolinska University Hospital, Stockholm, Sweden; 2 Department of Molecular Medicine and Surgery, Karolinska Institutet, Stockholm, Sweden; 3 Division of Radiology, Department for Clinical Science, Intervention and Technology (CLINTEC), Karolinska Institutet, Stockholm, Sweden; 4 Department of Oncology-Pathology, Karolinska Institutet, Stockholm, Sweden; 5 Department of Oncology, Södersjukhuset, Stockholm, Sweden; 6 Department of Clinical Science and Education, Karolinska Institutet, Södersjukhuset, Stockholm, Södersjukhuset, Stockholm, Sweden; 7 Department of Nuclear Medicine and Medical Physics, Theranostics Trial Center, Karolinska University Hospital, Stockholm, Sweden

**Correction: EJNMMI Research (2026) 16:96**

**https://doi.org/10.1186/s13550-026-01472-2**

Following publication of the original article, the authors identified an unintended methodological error in the application of the dynamic bladder model used for calculation of the time-integrated activity coefficient (TIAC) for the urinary bladder contents.

After correction of the methodological error and recalculation of the dosimetry, the mean absorbed dose to the urinary bladder wall increased from 100 µGy/MBq to 197 µGy/MBq, while other absorbed doses remained relatively constant.

As a direct consequence, the effective dose increased, from 11 µSv/MBq to 19 µSv/MBq. Despite this increase the revised doses remains within the lower range of values reported for other GRPR targeting radioligands presented in Table 1 in the published article.

The article has since been updated to correct for these errors. The authors thank you for reading this erratum apologize for any inconvenience caused.

